# Remarkable response to pembrolizumab with platinum‐doublet in PD‐L1‐low pulmonary sarcomatoid carcinoma: A case report

**DOI:** 10.1111/1759-7714.13890

**Published:** 2021-02-19

**Authors:** Hirokazu Taniguchi, Shinnosuke Takemoto, Mutsumi Ozasa, Noritaka Honda, Takayuki Suyama, Yasuhiro Umeyama, Yosuke Dotsu, Takumi Nakao, Kojima Tomohito, Hiroshi Gyotoku, Hiroyuki Yamaguchi, Taiga Miyazaki, Noriho Sakamoto, Yasushi Obase, Minoru Fukuda, Junya Fukuoka, Hiroshi Mukae

**Affiliations:** ^1^ Department of Respiratory Medicine Nagasaki University Graduate School of Biomedical Sciences Nagasaki Japan; ^2^ Molecular Pharmacology Program and Department of Medicine Memorial Sloan Kettering Cancer Center New York New York USA; ^3^ Department of Pathology Nagasaki University Graduate School of Biomedical Sciences Nagasaki Japan; ^4^ Department of Gastroenterology and Hepatology Nagasaki University Hospital Nagasaki Japan; ^5^ Department of Infectious Diseases Nagasaki University Graduate School of Biomedical Sciences Nagasaki Japan; ^6^ Clinical Oncology Center Nagasaki University Hospital Nagasaki Japan

**Keywords:** immunotherapy, programmed death‐ligand 1, pulmonary sarcomatoid carcinoma

## Abstract

Pulmonary sarcomatoid carcinoma (SC) is an aggressive subtype of lung cancer that exhibits resistance to cytotoxic chemotherapy. Although programmed cell death 1 (PD‐1) inhibitors have been reported to show antitumor effects in patients with high programmed death‐ligand 1 (PD‐L1) expressing SC, the efficacy of combined therapy with PD‐1 inhibitor plus cytotoxic chemotherapy has not previously been clarified. We herein report a case of SC with low expression of PD‐L1 and few pre‐existing tumor‐infiltrating lymphocytes which showed a remarkable response to pembrolizumab plus cytotoxic chemotherapy as first‐line treatment. Our findings suggest that combined treatment might enhance the immunogenic response, even in immunologically ignored SCs.

## Graphical abstract

Pulmonary sarcomatoid carcinoma (SC) is an aggressive subtype of lung cancer that exhibits resistance to cytotoxic chemotherapy and the efficacy of combined therapy with PD‐1 inhibitor plus cytotoxic chemotherapy as first‐line treatment has not previously been clarified. Here we report a case of SC with low expression of PD‐L1 and few pre‐existing tumor‐infiltrating lymphocytes which showed a remarkable response to pembrolizumab plus cytotoxic chemotherapy. Our findings suggest that treatment might enhance the immunogenic response, even in immunologically ignored SCs.

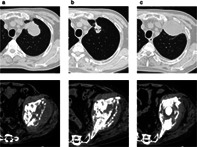

## INTRODUCTION

Pulmonary sarcomatoid carcinoma (SC) is a rare type of non‐small cell lung cancer (NSCLC) with a poor prognosis due to rapid tumor growth, early metastasis, and resistance to platinum‐based standard chemotherapy.^1^, ^2^ Novel therapeutic strategies for the treatment of SC are urgently needed to improve clinical outcomes.

Immune checkpoint inhibitors (ICIs) that block programmed cell death 1 (PD‐1) and programmed death‐ligand 1 (PD‐L1), either as single agents or in combination, have led to revolutionary treatments for NSCLC.^3^, ^4^, ^5^, ^6^, ^7^ A recent retrospective study suggested that single use of PD‐1 inhibitor was effective in SC patients as a second‐ or third‐line treatment.^8^ However, the efficacy of PD‐1 inhibitor with platinum‐doublet, especially as first‐line treatment, has not yet been elucidated.

Here, we report a patient with SC who showed a remarkable tumor response to PD‐1 inhibitor, pembrolizumab with carboplatin (CBDCA) plus pemetrexed (PEM), regardless of PD‐L1 tumor proportion score (TPS) 1%, and few tumor‐infiltrating lymphocytes (TILs) and few PD‐1^+^ immune cells were observed in tumor biopsy samples.

## CASE REPORT

**FIGURE 1:**
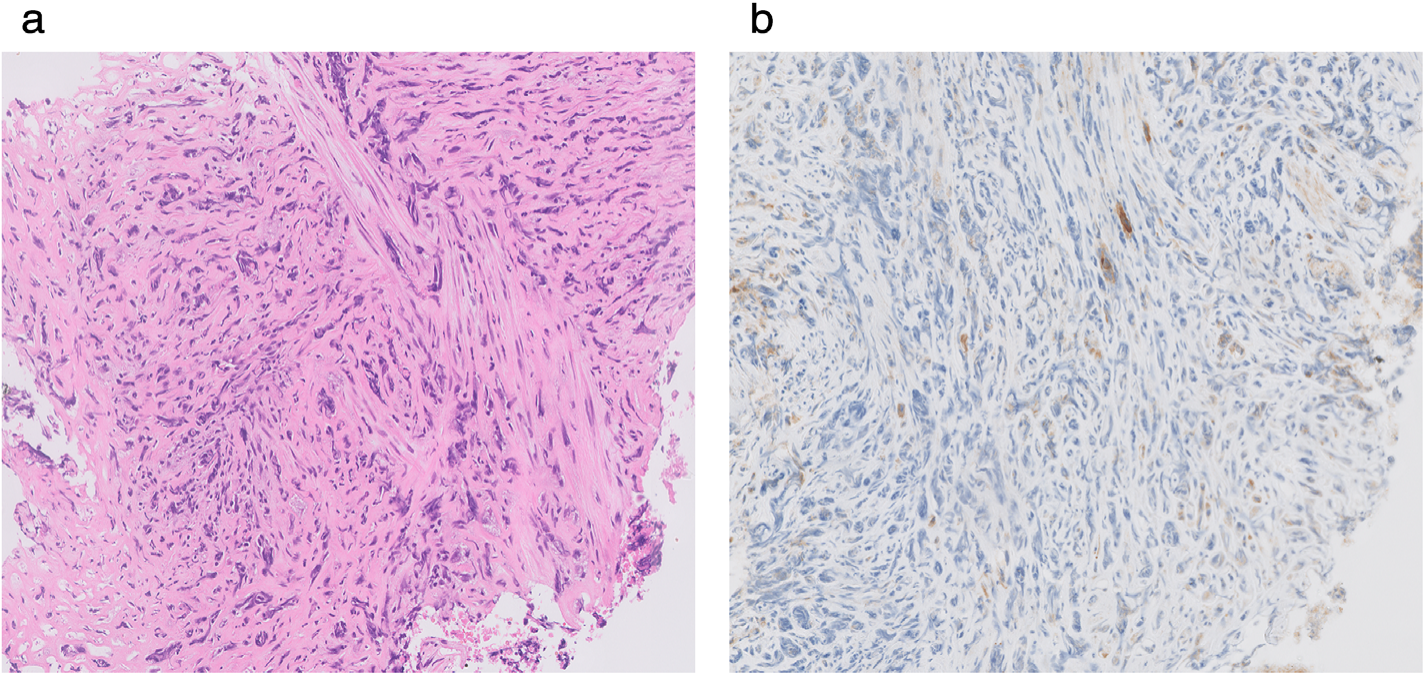
Pathological analysis of the specimen from primary lung cancer site taken from a patient with pulmonary sarcomatoid carcinoma. (a) Hematoxylin and eosin stain, 50×. (b) Immunohistochemical examination showed that 1% of the tumor cells expressed PD‐L1, 50 ×

A 65‐year‐old male patient presented to our hospital with a history of a cough and hip joint pain. Computed tomography (CT) revealed a mass lesion in the left upper lung lobe and a large mass in the left pelvis. Radiographic and pathological evaluations of the biopsy samples from the left lung and pelvic tumors led to a diagnosis of advanced SC with bone metastasis (cT4N0M1c, cStage IVB, UICC version 8). Immunohistochemistry (IHC) analysis of the biopsy sample from the lung tumor demonstrated 1% PD‐L1 expression on tumor cells (clone 22C3, Dako) (Figure 1(a),(b)), and no druggable driver mutations were observed.

**FIGURE 2:**
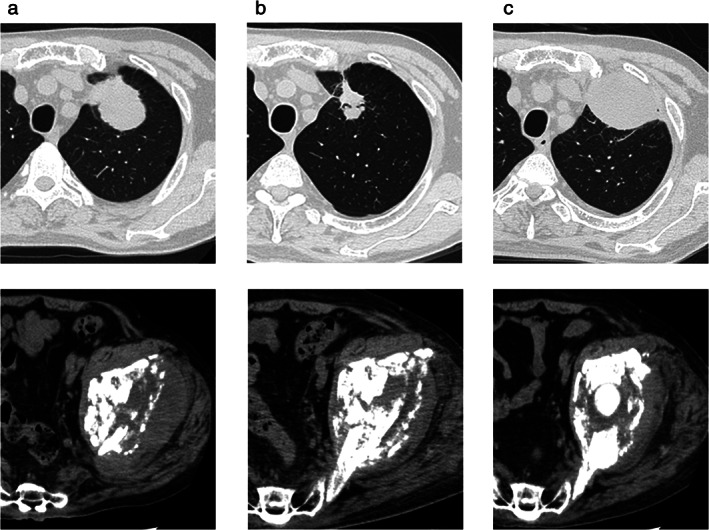
Chest and pelvis computed tomography (CT) scans in a patient with pulmonary sarcomatoid carcinoma. (a) Before treatment with pembrolizumab/carboplatin/pemetrexed, the size of the lung tumor was 50 mm in diameter. (b) After four courses of pembrolizumab/carboplatin/pemetrexed, the diameter of the lung tumor decreased to 26.4 mm. (c) A total of 84 days after initiation of treatment with prednisolone, the diameter of the lung tumor had increased to 62.3 mm. (upper, primary lung tumor; lower, metastasis site of left pelvis, respectively)

**FIGURE 3:**
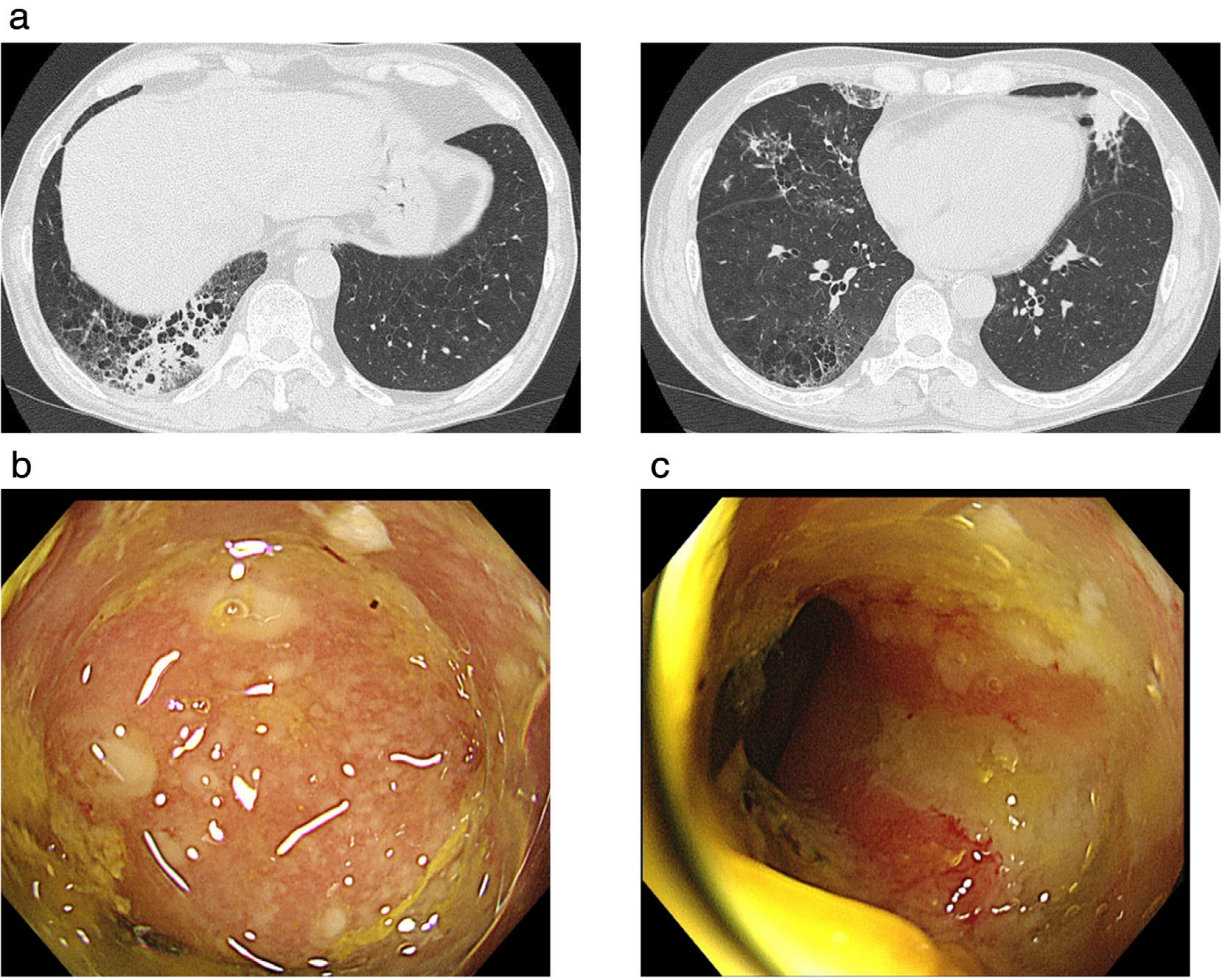
Image of immune‐related adverse events (iRAEs). (a) Chest computed tomography (CT) scans showing pneumonitis in the right lower lobe (left); the right middle and lower lobes, and the left lingular segment (right). (b and c) Colonoscopy revealed multiple ulcerations in the descending colon

The patient received four cycles of pembrolizumab (200 mg/bodyweight) with CBDCA (AUC 5) plus PEM (500 mg/m^2^) after palliative local radiotherapy (40 Gy/20 Fr.) to his left pelvis. The tumor in the left lung shrunk significantly and the left pelvic bone metastasis underwent ossification after four cycles of treatment (Figure 2(a),(b)). However, treatment was discontinued because the patient developed pneumonitis and colitis which were considered to be severe immune‐related adverse events (iRAEs) (Figure 3(a)–(c)). The patient commenced immunosuppressive treatment with 60 mg of prednisolone 38 days after the initiation of the fourth cycle of treatment. His pneumonitis and colitis fortunately improved; however, the tumor in his left lung had a significant regrowth 84 days after the initiation of prednisolone (Figure 2(c)), whereas the site of bone metastasis was stable, probably due to prior irradiation. After the iRAEs had subsided, the patient received docetaxel (60 mg/m^2^) plus ramucirumab (10 mg/kg) as second‐line treatment because the tumor size had evidently increased in size at that point. However, the mass in the left lung significantly increased after two cycles of docetaxel plus ramucirumab treatment (an increase in diameter from 93 mm before treatment to 125 mm after treatment). The patient chose not to receive third‐line therapy and continued with best supportive care due to a decrease in performance status.

**FIGURE 4:**
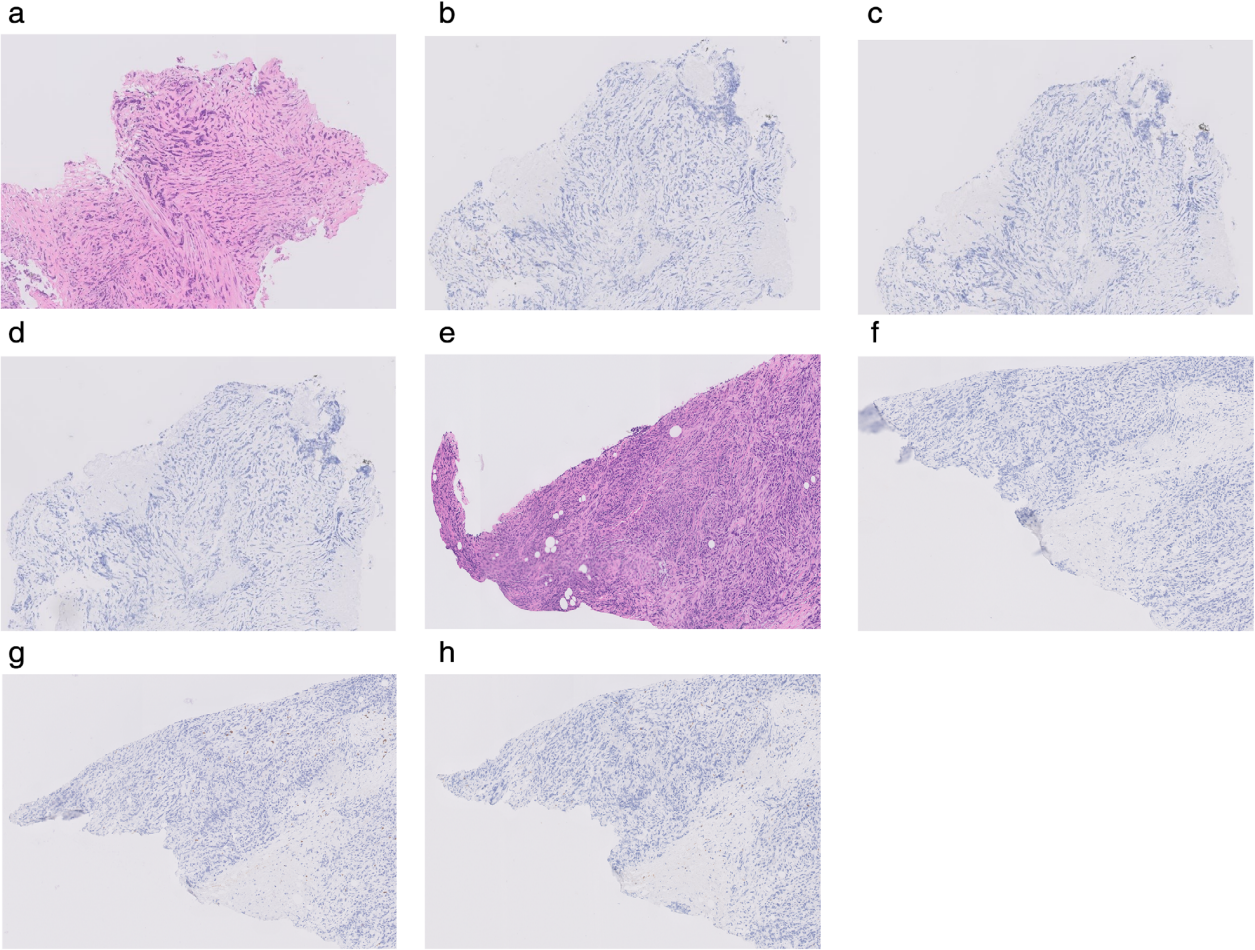
Immunohistochemical analysis of the specimen from primary lung cancer and metastasis site (left pelvis) taken from a patient with pulmonary sarcomatoid carcinoma. (a)–(d) the specimen from primary lung cancer. (a) Hematoxylin and eosin stain, 50×, (b) CD4^+^ T cells, 50×. (c) CD8^+^ T cells, 50×. (d) PD‐1^+^ cells, 50×. (e)–(h) The specimen from the site of metastasis (left pelvis). (e) Hematoxylin and eosin stain, 50×. (f) CD4^+^ T cells, 50×. (g) CD8^+^ T cells, 50×. (h) PD‐1^+^ cells, 50 ×

IHC review of the biopsy samples from the left lung and pelvic tumor at the time of diagnosis showed that there were few TILs including CD8^+^ T cells (NCL‐L‐CD8‐4B11, Leica Microsystems), CD4^+^ T cells (NCL‐L‐CD4‐1F6, Leica Microsystems), and PD‐1^+^ cells (NAT105, Abcam) in both biopsy samples obtained from the primary lung cancer site and metastatic site of the left pelvis (Figure 4(a)–(h)).

Written informed consent for the publication of this case report was obtained from the patient. This case study was approved by the Institution Review Board of the ethics committee of our institution (Approval #20122147).

## DISCUSSION

PD‐1 inhibitors have demonstrated novel therapeutic success by overcoming tumor‐induced T cell inhibition. CD8^+^ positive T cells are considered a critical component of antitumor immune response, and increased levels of CD8^+^ TILs have previously been reported to be associated with better outcomes in 552 patients with NSCLC.^9^ Pre‐existing CD8^+^ T cells distinctly located at the invasive tumor margin have been demonstrated to predict the response to ICIs in several types of cancers.^10^, ^11^ Expression of PD‐L1 on tumor cells has also been shown to be a predictive factor for the efficacy of PD‐1 inhibition in many solid tumors, including NSCLC.^6^, ^12^ Inflammatory cytokines such as IFNγ upregulate PD‐L1 expression in various cell types and TILs release IFNγ as an adaptive immune resistance.^13^ Thus, TILs or expression of PD‐L1 on tumors have been reported as predictive markers for the effectivity of PD‐1 inhibitors. A previous study reported that the response rate of PD‐1 inhibitors tended to be lower in the lower PD‐L1 expression cases in SCs.^8^

On the other hand, recent clinical trials showed that the addition of PD‐1 or PD‐L1 inhibitor with platinum‐doublet resulted in significantly longer overall and progression‐free survival (PFS) than placebo with platinum‐doublet across all categories of PD‐L1 expression in patients with NSCLC.^4^, ^5^ In addition to direct antitumor effects, a combination of PD‐1 inhibitor with cytotoxic chemotherapy has previously been reported to enhance the immunogenic response by the release of potentially immunogenic tumor antigens, promotion of the infiltration of CD8^+^ T cells, and increasing the ratio of cytotoxic lymphocytes to regulatory T cells^14^, ^15^, ^16^ even in patients with NSCLC that lacked pre‐existing T cell infiltrates or low PD‐L1 expression, as in the present case.

There are some limitations in the case reported here. The samples that were evaluated might not have captured the whole tumor microenvironment completely because they were obtained by needle biopsy, although the same results were obtained from two other tumor sites. Another limitation is that the tumor shrinkage might partially have been due to the abscopal effect wherein local irradiation can reduce the size of the nonirradiated site mediated by the immune system.^17^

In conclusion, pembrolizumab with CBDCA plus PEM demonstrated antitumor activity in SC with low PD‐L1 expression and few TILs. Further studies using a large cohort are needed to elucidate whether this therapeutic approach contribute to the survival of patients with SC across all categories of PD‐L1 expression and pre‐existing TILs.

## CONFLICT OF INTEREST

The authors report no conflicts of interest related to this work.
